# All-hazards dataset mined from the US National Incident Management System 1999–2014

**DOI:** 10.1038/s41597-020-0403-0

**Published:** 2020-02-21

**Authors:** Lise A. St. Denis, Nathan P. Mietkiewicz, Karen C. Short, Mollie Buckland, Jennifer K. Balch

**Affiliations:** 1 Earth Lab, 4001 Discovery Drive Suite S348–UCB 611, University of Colorado-Boulder, Boulder, Colorado, 80309 USA; 2 Cooperative Institute for Research in Environmental Sciences, 216 UCB, University of Colorado-Boulder, Boulder, Colorado, 80309 USA; 3 Department of Geography, GUGG 110, 260 UCB, University of Colorado-Boulder, Boulder, Colorado, 80309 USA; 4 USDA Forest Service, Rocky Mountain Research Station, 800 Beckwith Ave., Missoula, MT 59801 USA

**Keywords:** Socioeconomic scenarios, Natural hazards

## Abstract

This paper describes a new dataset mined from the public archive (1999–2014) of the U.S. National Incident Management System/Incident Command System Incident Status Summary Form (a total of 124,411 reports for 25,083 incidents, including 24,608 wildfires). This system captures detailed information on incident management costs, personnel, hazard characteristics, values at risk, fatalities, and structural damage. Most (98.5%) of the reports are fire-related, followed in decreasing order by *other*, hurricane, hazardous materials, flood, tornado, search and rescue, civil unrest, and winter storms. The archive, although publicly available, has been difficult to use due to multiple record formats, inconsistent free-form fields, and no bridge between individual reports and high-level incident analysis. Here, we describe this improved dataset and the open, reproducible methods used, including merging records across three versions of the system, cleaning and aligning with the current system, smoothing values across reports, and supporting incident-level analysis. This integrated record offers the opportunity to explore the daily progression of the most costly, damaging, and deadly events in the U.S., particularly for wildfires.

## Abstract (summary)

**Table:** 

Measurement(s)	wildfire • cyclone • hazardous materials • flood • tornado • search and rescue • civil unrest • storm
Technology Type(s)	digital curation
Factor Type(s)	year
Sample Characteristic - Location	United States of America

Machine-accessible metadata file describing the reported data: 10.6084/m9.figshare.11663076

## Background & Summary

There has been a steady rise in the occurrence of billion-dollar disasters in the United States (U.S.) since the 1980s, with the past three years (2016–2018) each setting historic highs. The average number of billion-dollar disasters across this period has more than doubled (from 6 to 15 events per year^1^). Further, cumulative costs in 2017 set a new annual record of $306.2 billion^2^. There is evidence that the frequency and magnitude of natural disasters is changing, with some extreme weather events linked to anthropogenic climate change^3^, such as the intensity of tropical storms^4^. Further, within just the past few decades, the area burned by wildfires in the western U.S. has increased at least threefold^5–8^, with a strong climate change influence for forest systems^9^. This is a critical moment to develop new methods and data sources to help understand the interrelationships between the physical and environmental characteristics of a hazard, the effectiveness of incident management strategies, and the societal impacts of these large-scale events.

Wildfires and hurricanes are two natural hazards that cause significant societal impacts, and require costly and complex incident response. In the last two years, damages from wildfires alone have exceeded $40 billion^1^ and current suppression costs average $2-3 billion each year^10^. For example, in the last two years, California has seen the largest, costliest, and deadliest fires in state history. More than 33,000 homes burned and 130 people died in wildfires in the past two years (www.iii.org/fact-statistic/facts-statistics-wildfires#top). Another striking impact is that of hurricanes between 2016 and 2018, when six separate billion-dollar hurricanes made landfall in the U.S. with an inflation-adjusted total loss of $329.9 billion and 3,318 fatalities^1^. When these events turn into disasters with large societal impacts, they necessitate a government-coordinated response with critical documentation about how the event is unfolding and threats to life and property.

The Incident Command System Incident Status Summary Form 209 (ICS-209) captures a unique perspective across an important population of hazard events. It is intended specifically for significant incidents that operate for an extended duration, compete with other incidents for scarce resources, or require significant mutual aid/additional support and attention^11^. Further, the ICS-209 is intended for use when an incident becomes significant enough to merit special attention, such as those that attract media attention, or when there is increased threat to public safety. Over 98% of the incidents in the dataset are wildfires. Although only 1–2% of wildfires become large incidents, they account for approximately 85% of total suppression costs and upwards of 95% of total acres burned each year^12^. The ICS-209 captures the best in-the-moment observations about the current and forecasted status of the hazard, current resources assigned, estimated costs, current and forecasted critical needs, and the societal and natural values currently at threat. These status summaries are required for each operational period of an incident response or when significant events warrant a status update. As a result, these reports offer a unique opportunity to study the relationship between hazard characteristics, incident response, and the societal impacts/threats incrementally across all phases of active response.

This paper describes the methods used to create and refine this dataset into a science-grade database. We provide a brief overview of the all-hazards dataset and then examine high-level spatial and temporal trends for wildfires across several key variables captured by these reports. We compare these values with a larger population of wildfires in the U.S. We then look in detail at relationships between these variables for the 2013 Rim Fire, a large catastrophic event, for insight into how these values might be combined with other sources of data such as satellite-derived datasets. We conclude by identifying the key opportunities for use of this dataset and how this open-source solution could be extended in the future.

## Methods

### Data source

Historical data from the ICS-209s are archived by the National Wildfire Coordinating Group (NWCG) on the Fire and Aviation Management Website (FAMWEB) in two formats: an HTML version of the forms (2001 to 2013), published at (https://fam.nwcg.gov/fam-web/hist_209/report_list_209), and the raw data, published annually on the FAMWEB homepage (https://fam.nwcg.gov/fam-web). We downloaded and compared the data from both sources and determined that the overlapping records were identical, yet the raw data covered a wider timespan, was more complete and included all records up to the most recent completed year. All data in the ICS-209-PLUS dataset are sourced from the raw data files, and the dataset is designed so that it can be extended as new data is released. We manually downloaded the raw data from FAMWEB, saving each table in Excel format. The data span three separate versions that we will refer to as Historical System 1 (HIST1) from 1999 to 2002, Historical System 2 (HIST2) from 2001 to 2013, and the current version (CURRENT) from 2014 to the present. The input tables for each of these versions are summarized in Table 1 below. The ICS-209-PLUS dataset is currently truncated in 2014 due to missing tables and data duplication issues in 2015 through 2017. If these issues can be resolved, we will extend the dataset to include records from the most recent years. We describe the handling of data from overlapping years in the *Purging Duplicate and Erroneous Records* section below.

**Table 1:** Input Table Names.

Table Content	System Version	Table Name(s)
Fire Complex Record	Historical System 2*	IMSR_IMSR_209_INCIDENT_COMPLEX
	Current System	SIT209_HISTORY_INCIDENT_COMPLEX_ASSOCS
Incident Record	Current System	SIT209_HISTORY_INCIDENTS
Daily Situation Report	Historical System 1	IMSR_INCIDENT_INFORMATIONS
	Historical System 2	IMSR_IMSR_209_INCIDENTS
	Current System	SIT209_HISTORY_INCIDENT_209_REPORTS
Current Resources	Historical System 1	IMSR_INCIDENT_RESOURCES
	Historical System 2	IMSR_IMSR_209_RESOURCES
	Current System	SIT209_HISTORY_INCIDENT_209_RES_UTILIZATIONS
Structural Information	Historical System 1	IMSR_INCIDENT_STRUCTURES
	Historical System 2	IMSR_IMSR_209_INCIDENT_STRUCTURES
	Current System	SIT209_HISTORY_INCIDENT_209_AFFECTED_STRUCTS
Casualties & Illnesses	Current System	SIT209_HISTORY_INCIDENT_209_CSLTY_ILLNESSES
Life Safety Management	Current System	SIT209_HISTORY_INCIDENT_209_LIFE_SAFETY_MGMTS
Suppression Strategies	Current System	SIT209_HISTORY_INCIDENT_209_STRATEGIES
Lookup Tables	Historical System 1, Historical System 2	IMSR_LOOKUPS, IMSR_STATES
	Current System	SIT209_HISTORY_SIT209_LOOKUP_CODES, COMMONDATA_NWCG_UNITS, COMMONDATA_STATES

*2010 to 2013 only.

### History of ICS

The ICS-209 form, commonly referred to as a *sitrep*, is part of the U.S. National Incident Management System/Incident Command System (NIMS/ICS). The earliest implementation of ICS was developed by the U.S. Forest Service (USFS) following a devastating fire in California in 1970 that claimed 16 lives, destroyed over 700 structures and burned over 500k acres. Numerous communication and coordination issues hampered the effectiveness of the agencies involved, resulting in a congressional mandate requiring the USFS to design a new system to facilitate interagency coordination and to support the allocation of suppression resources in dynamic, multi-fire situations^13–15^. The USFS worked in collaboration with California state agencies to produce FIRESCOPE (FIrefighting RESources of California Organized for Potential Emergencies) with two key components: the Incident Command System (ICS) and the Multi-Agency Coordination System (MACS^13^). By 1981, FIRESCOPE was used by agencies throughout Southern California and was adapted for non-fire use. In parallel with this effort, the NWCG adopted and revised the FIRESCOPE ICS documentation to create the National Interagency Incident Management System (NIIMS) Incident Command System Operational System Description (ICS 120-1) - a document that was collectively maintained by CalFire and NWCG. This document later served as the basis for NIMS ICS^15^. Following the 2001 September 11th terrorist attacks in the U.S., the Department of Homeland Security (DHS) was formed, and on February 28, 2003, President George W. Bush issued Presidential Directive-5^16^ calling for the establishment of a single, comprehensive national incident management system, which became NIMS.

### NIMS ICS

The NIMS was issued in March 2004 to enable responders at all jurisdictional levels and disciplines to work together more effectively by establishing a single, comprehensive national incident management structure^14,15^. In 2005, there was a push to institutionalize the use of ICS across the entire response system and by 2006, federal funding for state, local and tribal grants was tied directly to compliance with the NIMS^15^. The NIMS/ICS built upon existing incident management best practices including ICS and MACS. It fully delineates standardized command and control structures and procedures designed to support interoperability among jurisdictions and across disciplines as the complexity of a response effort increases. The planning function is centralized within ICS with information captured during each operational period flowing up to the tactical and strategic planning level^14^.

### The ICS 209 incident status summary

The Federal Emergency Management Association (FEMA) describes the purpose of the ICS-209 as follows: “*The ICS 209 is used for reporting information on significant incidents … The ICS 209 contains basic information elements to support decision making at all levels above the incident to support the incident*. *Decision makers may include the agency having jurisdiction*, *but also all multiagency coordination system (MACS) elements and parties*, *such as cooperating and assisting agencies/organizations*, *dispatch centers*, *emergency operation centers*, *administrators*, *elected officials*, *and local*, *tribal*, *county*, *State*, *and Federal agencies*.*”*^11^. The ICS-209 is described as providing a “snapshot in time”, capturing the most accurate and up-to-date information available at the time of preparation. The form is typically completed by the Situation Unit Leader or Section Planning Chief within the Incident Management Team, but may also be completed by a local dispatcher or another staff member when necessary. Reports are logged for each operational period or when information becomes outdated in a quickly evolving incident. Each report describes current characteristics of the hazard, current environmental conditions, current and projected incident management costs, details about specific resources assigned to the incident, critical resource needs, a description of structural and life safety threats, an ongoing accounting of injuries, fatalities, damages, and the projected incident management outlook.

The format and content of the ICS-209 has evolved over time in parallel with efforts to adapt the form for all-hazards use. Our inspection of records identified new fields added in 2004, when the system was incorporated into NIMS, and again in 2007 to support all-hazards reporting. It is important to note that the use of NIMS/ICS was not mandatory on large incidents until fiscal year 2006^15^, and so there may be significant gaps in reporting prior to this date. Any time-series analysis exploring trends in the data must acknowledge this limitation. Additionally, the ICS-209-PLUS dataset is based on the published data and may exclude records containing sensitive information.

The raw data are published in three separate formats. The original format, Historical System 1 (HIST1), spans 1999 to 2002 and includes basic incident information, start location, personnel usage, and total structures damaged/destroyed. The second format, Historical System 2 (HIST 2), was introduced in 2001 and captures a broader set of information related to the hazard including incident complexity, fire behavior, fuels, and local weather. It contains freeform narrative text fields to capture projected risk to communities, resources at risk, critical resource needs, planned actions, and projected hazard movement/spread. Additional societal impact values include injuries, fatalities, evacuations in progress, and estimates of structures threatened. The current system format was released in 2014. Tighter standardization of values on the form resulted in cleaner categorical data. Major changes include an expansion of formats for capturing point-of-origin data, expanded functionality for tracking casualties and illnesses, and expanded functionality for tracking life safety management. Table 2 provides a high-level summary of data elements in the ICS-209-PLUS dataset. Refer to^17^ for a description of individual fields in the ICS-209-PLUS *sitrep* table. Additionally, the FEMA ICS-209 Form^11^ describes the intended use of each field in the ICS-209 form and whether or not field is required.

**Table 2:** Description of Data Elements by Type on ICS-209 Incident Status Form.

Information Type	Data Elements
Incident Reporting Detail	Incident Name, Incident Number, Reporting Time Period, Report Status (Initial, Update, Final), Approval and Routing Information
Current Incident Status	Incident Commander(s), Incident Management Organization, Level of Complexity, Percent Contained/Completed, Estimated Costs to Date, Current Resources (Personnel and Equipment currently in use), Agencies Involved, Additional Cooperating and Assisting Agencies, Fire Complex Details
Hazard Description & Conditions	Incident Type, Cause, Start Date/Time, Location, Current Area Involved, Materials/Hazards Involved, Fuels, Fuel Conditions, Fire Behavior, Current & Forecast Weather
Projected Outlook & Needs	Critical Resource Needs, Current and Projected Weather/Conditions, Projected activity/movement/escalation or spread, Strategic Objectives, Planned Actions, Projected Final Size, Projected Final Costs, Projected Containment Date, Projected Demobilization Date
Societal Impacts	Structures threatened/damaged/destroyed, Values at Risk, Injuries, Fatalities

### The ICS-209 situation report & related tables

The situation report or *sitrep* table contains the majority of fields in the *Incident Status Summary*. In addition to this, all three versions have a *resources* table that tracks personnel and equipment by response agency. The earliest system (Historical System 1) tracks the fewest number of resource types (*Crew 1–3*, *Helicopter 1–3* and *Overhead Personnel*) with estimated *Total Personnel* stored directly in the *sitrep* table. Later versions expand on these resource types and add a text field to the *sitrep* table to capture additional cooperating agencies. We pivot the *resources* tables by agency to calculate *Total Personnel* for each *sitrep*. All three versions also have a *structures* table that tracks the number of structures threatened, damaged, or destroyed by commercial, residential, and outbuilding structure types. We pivot the structures table by structure type to calculate totals related to structures threatened, damaged, or destroyed for each *sitrep*. Both historical versions track injuries and fatalities directly in the *sitrep* table, whereas the current system tracks this data in the *Casualties and Illnesses* table. This new table introduces a broader range of individual impacts including the number of people missing, trapped, evacuated, sheltering in place, in temporary shelters, immunized, and quarantined. Additionally, the *Life Safety Managements Table* in the current system keeps track of incident management activity related to mass notifications, area restrictions, evacuations, immunizations, quarantine and sheltering-in-place. We use the *Casualties and Illnesses* table to calculate the number of injuries and fatalities and evacuation status for each *sitrep*. This could be expanded in future releases to track a broader range of life safety threat and current response status. Finally, there is an annual *Lookup Codes* table containing standardized field values. These values have remained fairly consistent with new values added over time. Current code values are summarized in the standard codes reference^14^. Any modifications to existing values are discussed in the *Transforming Standardized Fields* section below.

There are two parent tables related to the *sitrep* table: an *incidents* table and a *complexes* table. The complexes table was added in 2010. A complex incident is defined as *two or more individual incidents located in the same general area which are assigned to a single incident commander or unified command*^15^. The complex record clusters all fires and *sitreps* associated with a fire complex under the same incident number, capturing individual fire names, suppression strategy, current containment percentage and estimated costs to date. The current version also includes the current area for individual fires within the complex. This information is missing in both historical versions of the system. We used data that was manually compiled and verified to derive a *complex associations* table for wildfires between 1999 and 2013 and we produce a similar table for 2014 to describe the relationship between fire complexes and individual fires. These supplementary tables are described in section 7 below. Finally, the current version has an incidents table that contains basic incident level information including discovery date, cause, area, location, and estimated cost to date. Concatenated versions of the original complex and incident tables are included in the dataset (Table 1), but we did not clean or modify these tables. We created a new *Wildfire Incident Summary Table*^17^ derived from the cleaned and smoothed values in the Wildfire *sitreps* table. This new incident level table contains additional incident level statistics that enhance the research value of the dataset. This new table is described at the end of the methods section.

### Open/reproducible framework

We produced the ICS-209-PLUS dataset using principles of open and reproducible science^18–20^. All data source files and the final ICS-209-PLUS dataset are archived online^17^. The python source code for the ICS-209-PLUS creation^21^ and R code used for spatial database creation and the creation of all figures and tables^22^ are publicly available. Our aims are twofold: to provide transparency to the methods and assumptions used to produce the final dataset and to provide a framework for others to adapt or expand upon the dataset. The code is written in Python using the Numpy and Pandas data science libraries. We were unable to automate the downloading of the raw data from FAMWEB and so our code assumes all relevant tables (Table 1) are downloaded to the corresponding annual directories beforehand.

We accomplished several key objectives in this first release. First, we aligned data elements and standardized values across both historical versions with the current data model. This allows for seamless comparison of records across the entire time-period. Secondly, due to the free-form nature of the fields and limited mechanisms enforcing data entry standards, the original data is notoriously messy and difficult to use. The scripts are designed to automate as much of the cleaning and formatting as possible, improving the overall consistency of the dataset. It is also designed to support the manual updates identified in the process of producing the dataset. This is important for several reasons. It allowed us to easily incorporate updates for fields such as *Latitude* and *Longitude* that were deemed critical for dataset use. It also provided a framework for incorporating the cleaning efforts and refinements from the 209 dataset curated by Karen Short. Finally, we connect the ICS-209-PLUS dataset with the Fire Program Analysis fire-occurrence database (FPA FOD^23^) enabling linkage with fire perimeter data and final fire statistics. The following sections detail how the dataset is produced from the merging of the original source files to the creation of the *Wildfire Incident Summary* table and external linkages.

### Producing the ICS-209-PLUS dataset

The ICS-209-PLUS dataset is produced by a series of Python scripts that first consolidates the annual files for each of the tables across the three versions of the system (Table 1). Each version of the *sitrep* table is then cleaned and prepared for the merge. This includes general cleaning and formatting for each field, field-level updates to correct known errors, and deletion of duplicates/erroneous records. Each of the related tables are pivoted and totals are calculated for personnel, aerial equipment, structures threatened, structures damaged, structures destroyed, injuries, fatalities, and evacuation status (2014+) for each situation report. These totals are joined into the situation report and then columns across the three versions are aligned and concatenated. Once the data has been consolidated into a single dataset, individual fields are cleaned and smoothed, filling missing values and adjusting values where appropriate. This finalized version is then used to produce an all-hazards dataset (ICS-209-PLUS All-Hazards), and a wildfire dataset (ICS-209-PLUS WF). The wildfire dataset is composed of two tables: all the wildfire daily status summaries and an incident level summary record. The *Wildfire Incident Summary* contains high-level statistics that are useful from a research standpoint.

### Cleaning and formatting the individual datasets

The script cleans each version of the *sitrep* table prior to the merge. This is necessary to deal with subtle differences between each version. Unique identifiers are constructed within the historical datasets to separate out individual fire events and to group related incidents together. We clean and standardize values for each historical version so that they merge smoothly into the final dataset. Once this preliminary cleaning is complete, members of the historical dataset are compared with a refined version of the record and *sitreps* that are not members of this refined set are archived to a *deleted sitreps* table (described later).

### Creating unique incident and fire identifiers

The *Incident Number* field is meant to uniquely identify an incident, but there are multiple issues with this field, particularly in the historical datasets. In some instances, incident numbers are incomplete or they are re-used from year to year, resulting in *sitreps* for multiple incidents being grouped together as a single incident. Splitting them based on year is problematic because some fires, particularly in the southeastern United States span the annual boundary or have a final report filed in the next year. There are also instances where Incident Name and point of origin are distinctly different but share the same incident number. Conversely, there are incidents in the current version that have the same incident number, but are split across multiple unique system identifiers. Finally, there are instances where fires are incorporated into a fire complex and the *Incident Number* changes to that of the fire complex. We solved these issues by creating two concatenated ID fields: the *Fire Event ID* and the *Incident ID*. The *Fire Event ID* is used to identify individual wildfires regardless of whether they are managed as part of a larger fire complex. The *Incident Id* is used to group all sitreps related to an incident response, clustering related situation reports that are related but may differ in terms of the *Incident Number* and or the *Incident Name*.

### The fire event ID

The *Fire Event ID* is a concatenation of the *Start Year* and the *Incident Number* fields followed by a sequence number (default = 1). The *Start Year* separates instances where the *Incident Number* is re-used from year-to-year. We manually scanned sitreps in both historical versions sorted by *Incident Number*, *Incident Name*, *Discovery Date*, and the report date to identify records that needed to be split. For example, Incident Number “AR-ARS-D2” was assigned to three separate incidents starting in different locations at different times (Table 3). We split them by adjusting the sequential variable for Dierks to 2 and Red Barn to 3.

**Table 3:** Example of Fire Event ID splitting three separate Wildfires sharing same Incident Number.

Incident Name	Location	Coordinates	Discovery Date	Fire Event ID
Vandervoort	3 miles NE of Vandervoort AR	33.134167, −93.858333	2011-04-03 21:41:00	2011|AR-ARS-D2|**1**
Dierks	7 miles NE of Dierks	34.145833, −93.895	2011-04-03 15:12:00	2011|AR-ARS-D2|**2**
Red Barn	East of Cowlingsville	33.869167, −94.087778	2011-09-10 15:30:00	2011|AR-ARS-D2|**3**

### The incident ID

The *Incident ID* is a concatenation of the *Start Year*, the final *Incident Number*, and the final *Incident Name*, such that multiple fires can be grouped together if they are later incorporated into a larger response. This information is missing in the historical datasets, but was manually compiled and verified by co-author Karen Short over time during the compilation of the Fire Program Analysis fire-occurrence database (FPA-FOD^23^). As a United States Forest Service employee, she was able to compare incident information with other internal sources to piece together relationships between individual fires and fire complexes and to purge duplicate and erroneous situation reports across the two historical records. We use this cleaned version of the sit-209 records, referred to as the Short master list^17^, as a definitive reference such that this table is used to create the *Incident ID* for all historical sitreps and determines which records should be deleted.

The example below is taken from the 2006 Boundary Complex Wildfire. It illustrates how *Incident ID* is used to group related fires together (Table 4) while preserving the original values. The complex includes the following individual fires: Elkhorn 2, Lost Lake, Deer, Thicket, Chuck, East Elk, North Elk, and Knapp 2, all under *Incident ID* 2006_ID-SCF-006336_BOUNDARY COMPLEX. The *Fire Event IDs* are included at the fire right to illustrate how the *Incident ID* allows for multiple physical fires to be grouped together as a single response, whereas the *Fire Event ID* provides a unique identifier for the physical fire event.

**Table 4:** Multiple Fires Grouped Within 2006_ID-SCF-006336_BOUNDARY COMPLEX Wildfire.

Incident Number	Incident Name	# sitreps	Start	Fire Event ID
ID-SCF-006336	Boundary	8	8/21	2006|ID-SCF-006336|1
ID-SCF-006336	Boundary Complex	39	8/8	2006|ID-SCF-006336|1
ID-SCF-6245	Elkhorn2	1	8/9	2006|ID-SCF-6245|1
ID-SCF-6349	Lost Lake	2	8/8	2006|ID-SCF-6349|1
ID-SCF-6369	Deer	2	8/31	2006|ID-SCF-6369|1
ID-SCF-6373	Thicket	1	8/7	2006|ID-SCF-6373|1
ID-SCF-6415	Chuck	3	8/9	2006|ID-SCF-6215|1
ID-SCF-6494	East Elk	1	8/21	2006|ID-SCF-6494|1
ID-SCF-6496	North Elk	2	8/21	2006|ID-SCF-6496|1
ID-SCF-6554	Knapp #2	1	9/7	2006|ID-SCF-6554|1
ID-SCF-6554	Knapp 2	4	9/7	2006|ID-SCF-6554|1

### General field level cleaning

We used the Python data science tools to inspect values contained in each column across the three versions to determine what actions were needed to clean and prepare for the merge. Many columns had standardized values, but contained extraneous characters or inconsistencies. The script uses regular expressions to standardize values for fields like *GACC Priority*, *Dispatch Priority*, *Percent Containment*, *Containment Date*, and *Incident Management Team Type* fields. Once these values are standardized, they are linked to corresponding values in the lookup code tables. The script also removes all linefeeds and hidden characters from text fields to make viewing and processing the fields easier. Values such as “N/A”, “same”, or “none” and redundant values are deleted from the consolidated text fields. The script fixes any obvious date errors (e.g., year values of 1901 instead of 2001) and applies consistent formatting across all date fields. All *Latitude* and *Longitude* values have been converted to decimal degrees. We cleaned and formatted most of the fields with the exception of weather variables and fuels. We determined that both of these fields would require extensive effort and fell outside the scope of this initial release.

Throughout the process, we identified individual values that were clearly an error and made some individual field level updates. These updates are limited and are incorporated into the general field cleaning function for each script. In the future, there is potential to maintain these updates as part of field level update table that could be loaded at runtime to automate individual field-level modifications. This would be an ideal solution to support ongoing update and maintenance of the dataset in the future but is beyond the scope of this initial release.

Finally, we use static information from the *Incidents Table* (SIT209_HISTORY_INCIDENTS) in the current version to fill missing values in the sitrep table. Values copied from the *Incidents Table* include: *Incident Name*, *Incident Number*, *Cause*, *Discovery Date*, *Incident Type*, *Short Location Description*, *Point of Origin City*, *Point of Origin State*, *County*, the *Legal Description* for the point of origin *(Township*, *Section* variables, *Range*, *Prime Meridian)*, and *Single/Complex Flag*. This patch was developed to repair a significant number of missing values in the pre-released 2016 sitrep table, including values used to build the *Incident Identifiers* and *Fire Identifiers* (*Incident Name*, *Incident Number*, *Discovery Date)*. Few values were changed in the 2014 dataset, particularly for columns with already high fill rates (~0–8 rows updated). There were modest improvements for *Point of Origin City* (152 rows filled) and *Legal Description* for point of origin (154 rows filled), but the overall fill rates remain the same. We applied the patch to the 2014 dataset so that when we publish later years, the data in the 2014 records will remain consistent over time.

### Transforming standardized fields

Standard values remained relatively consistent across the three versions, with new values added as the form was adapted for all-hazards use. The *Cause* and *Suppression Method Abbreviation* fields changed slightly from the historical to the new version and so we translated old values to equivalent new values (Table 5). A handful of Incident Types were eliminated in the current system. After careful consideration, we decided to keep the historical values for consistency and to prevent information loss. Prescribed burns (RX) and Wildfire for Resource Benefit have been included in the Wildfire datasets. We reclassified all values that were binary (yes/no) to boolean values (true/false) to make them consistent and to put them in a more standard database format.

**Table 5:** Standard Field Updates.

Field	Version	Original Value	New Value
Cause	Hist1, Hist2	**N** (No Description)	**O** (Other)
Complex	Hist2 Current	**Y/N** **S/C**	True/False
Evacuation In Progress	Hist2, Current	**Y/N**	True/False
Incident Type Abbreviation	Hist1, Hist2	**SAR** (Search & Rescue) **USR** (Urban Search & Rescue) **WFU** (Wildfire for Res Benefit) **RX** (Prescribed Burn) **OS** (Oil Spill) **LE** (Law Enforcement) **MC** (Mass Casualty) **STR** (Structure Fire) **BAR** (Burned Area Emergency Rehabilitation)	**SR/R (for Search & Rescue types)** All other values preserved to prevent data loss.
Suppression Method Abbreviation		**MM** (Monitor) **CC** (Confine) **PZ** (Point Zone Protection)	M (Monitor) C (Confine) PZP (Point Zone Protection)

### Cleaning and consolidating narrative text

Each version of the Incident Status Summary provides space for recording important observations from incident command. The earliest version of the report (Historical System 1) has only one *Narrative* field whereas later versions have multiple narrative text fields organized around the following topics: critical resource needs, current threats, projected incident movement and spread, weather, fuels, relevant conditions, and general remarks. Critical resource needs, current threats, and projected fire activity capture projected values at 12, 24, 48, 72, and greater than 72 hours from the current report. We consolidated these observations into one narrative field for each topic to manage the complexity of the dataset, eliminate redundancy, and to organize the observations for potential text mining and topic modeling efforts. Before consolidating, we clean each individual field to strip hidden characters, eliminate placeholder values (e.g. “n/a”, “same”, “none”) and eliminate duplicate values. A pipe ‘|’ character is used to separate observations. For example, the following values for the projected activity fields:

Projected Movement 12: *“Minimal fire movement due to lower temps higher RH and precipitation*.*”*

Projected Movement 24: *“Minimal fire movement due to lower temps higher RH and precipitation*.*”*

Projected Movement 48: *“Moderate fire activity is anticipated on Friday due to warming temps*, *falling RH*, *and wind*.*”*

Projected Movement 72: *“same”*

### Is consolidated into a single *projected activity narrative*

*“Minimal fire movement due to lower temps higher RH and precipitation|Moderate fire activity is anticipated on Friday due to warming temps*, *falling RH*, *and wind*.*”*

Table 6 summarizes the narrative text fields in the final dataset. The bold-faced fields are the newly consolidated fields that condense projected values into a single narrative summary. The version column identifies which versions populate this field.

**Table 6:** Narrative Fields in Final Dataset.

Field Name	Description	Version
ADDTNL_COOP_ASSIST_ORG_NARR	List of additional agencies not tracked in resources table cooperating on Fire.	HIST2, Current
**CRIT_RES_NEEDS_NARR**	Projected resource needs 12, 24, 48, 72 hours	HIST2, Current
**CURRENT_THREAT_NARR**	Current resources and values at risk.	HIST2, Current
HAZARDS_MATLS_INVOLVEMENT_NARR	Description of fuels and materials involved in fire.	HIST2, Current
LIFE_SAFETY_HEALTH_STATUS_NARR	Summary of current risk to life and health safety.	Current Only
MAJOR_PROBLEMS	Summary of any major problems	HIST2 Only
OBS_FIRE_BEHAVE	Description of current fire behavior	HIST2 Only
PLANNED_ACTIONS	Summary of planned actions	HIST2, Current
**PROJECTED_ACTIVITY_NARR**	Projected hazard activity 12, 24, 48, 72 hours	HIST2, Current
REMARKS	General Remarks Field	HIST1, HIST2, Current
SIGNIF_EVENTS_SUMMARY	Summary of significant events for the current operational period	HIST2, Current
STRATEGIC_NARR	Strategic Objectives and Strategic Discussion (current system only)	Current Only
**WEATHER_CONCERNS_NARR**	Current and projected weather outlook, consolidates weather observations from HIST2 into one parsable field.	HIST2, Current

### Linking to additional fire datasets

The co-authorship of this paper made linking with the Fire Program Analysis fire-occurrence database (FPA FOD^23^) a logical extension of the ICS-209-PLUS dataset. The FPA FOD database provides final determination for cause, containment and discovery dates, final acres, and connectivity to the Monitoring Trends in Burn Severity database (MTBS^24^). The MTBS database, in turn provides fire perimeter and burn severity data.

The *Incident ID* is used to join *Wildfire Incident Summary* records with records in the FPA FOD Extract file. The extract is an excel spreadsheet published as part of the dataset using the naming convention FOD_JOIN_{mmddyyyy}.xlsx. Matching records between the two datasets was an iterative process. At time of publication, all wildfire incidents occurring in United States and US Territories with a clearly defined point of origin larger than 1000 acres with corresponding record(s) in the FPA FOD database has been linked with 86% of incidents linking to at least one FPA FOD record. As we continue to clean and refine the dataset we will publish incremental updates to this file^17^.

As matches were identified between the two datasets, the Short Master List^17^, needed to be updated to maintain the relationships between fires and complexes in the FPA FOD dataset. This process for managing relationships between fires and complexes at the sitrep level for the historic dataset was unwieldy but difficult to modify given the significance of the Short master list in the merge and cleaning process for the historical data. We replaced this with a merge configuration file *FOD_CPLX_REF_2014*.*xlsx* for the current dataset (Table 7). This file is derived from the *Complex Associations Table* with additional rows added for complexes that are in the FPA FOD but not in the ICS-209-PLUS table. The table maintains relationship between fires and complexes and is used to map individual sitreps to the incident summary record for the fire complex and to the fire complex in the FPA FOD dataset.

**Table 7:** FOD Complex Cross Reference Table Description for Current System.

Column Name	Description
CPLX_INCIDENT_ID	Concatenated Incident ID for Fire Complex
CPLX_INC_IDENTIFIER	Internal Incident Identifier for table joins
MEMBER_INCIDENT_ID	Concatenated Incident ID for Member Fire in Complex
MEMBER INC_IDENTIFIER	Internal Incident Identifier for table joins
ICS_209_CPLX	Boolean – true if in complex associations table

### FPA FOD and MTBS fields

The majority of incidents in the ICS-209-PLUS dataset matched with a single record in the FPA FOD database (83%) but a small percentage (3%) were fire complexes associated with multiple records in the FPA FOD database. To balance the potential for multiple FPA FOD identifiers per incident with the more general case, we developed the following solution. We added the following fields to the Wildfire Incident Summary (Table 8). The values for the largest fire are used if there are multiple fires. The Cause field takes all unique values for cause. It is typically one, but will contain a list of values if there are multiple causes across a multi-fire incident. The discovery date is the earliest value in related FPA FOD records and the containment day is the latest. The *FOD_FINAL_ACRES* is the sum of all reported acres. The FOD_NUM_FIRES field is the number of fires linked to this incident and FOD_LIST stores information about all fires related to the incident.

**Table 8:** New fields from FPA FOD database added to Wildfire Incident Summary Table.

Column Name	Description
FOD_ID	Fire-occurrence database ID - largest fire if multiple fires
FOD_CAUSE_CODE	Cause code - numeric
FOD_CAUSE_DESCR	Cause description (e.g. Arson, Lightning, Debris, Campfire)
FOD_COMPLEX_NAME	Fire Complex Name
FOD_CONTAINMENT_DOY	Day of year for fire containment - minimum value if multiple fires
FOD_DISCOVERY_DOY	Day of year for discovery date - maximum value if multiple fires
FOD_FINAL_ACRES	Final size of fire (sum of acres if multiple fires)
FOD_LIST	List of FOD Fires related to this incident as key/value pairs
FOD_LATITUDE	FOD Latitude for point of origin (decimal)
FOD_LONGITUDE	FOD Longitude for point of origin (decimal)
FOD_NUM_FIRES	Number of FPA FOD records associated with incident
MTBS_ID	MTBS Identifier associated with incident – largest fire if multiples
MTBS_FIRE_NAME	MTBS Fire Name – largest fire if multiple fires

All FPA FOD records associated with an incident are stored in the *FOD Fire List* as a JSON object. This provides both a human-readable and machine parsable summary at the incident level. For example, the 1999 Arizona Jump Complex has three records in the fire-occurrence database and so the *FOD Fire List* contains three entries:

[{“**ID”**: 215365, “**MTBS_ID”**: “AZ3662411371319990528(JUMPSPRING)”,”**COORDS”**: (36.5928, −113.7352), “**CAUSE”**: “Lightning”, “**SIZE”**: 16816.0, “**DISC”**: 148, “**CONT”**: 154.0},

{“**ID”**: 215366, “**COORDS”**: (36.6216, −113.7172),”**CAUSE”**: “Lightning”, “**SIZE”**: 7.0, **“DISC”**: 148, “**CONT”**: 154.0},

{“**ID”**: 215369, “**COORDS”**: (36.96, −113.8158), “**CAUSE** “: “Lightning”, “**SIZE”**: 69.0,”**DISC”**: 149, “**CONT”**: 150.0}]

### Latitude/Longitude

Given the critical role point of origin data plays in geospatial analysis, we manually cleaned and inspected the point of origin coordinates, fixing obvious errors and providing estimates for missing or obviously erroneous values. The values in the earliest system (Historical System 1) were first converted from degrees and minutes to decimal format. We then mapped all the points, identifying those that fell outside of the United States and its territories. The most common issue was an incorrect numeric sign for latitude or longitude. The longitude was incorrect for 98.5% of the longitudes in the second historical system (Historical System 2) and 4% of the coordinates for 2014. Wherever possible, we used latitude/longitude values from the FPA-FOD Fire-occurrence database for missing and erroneous values. We then manually examined the remaining values that fell outside of the clipped boundaries individually. We used the information contained in other point of origin fields (e.g., the location description) to estimate latitude and longitude. For each of these estimated values, we set the *Lat/Long Update* flag to true and set the *Lat/Long Confidence* field to capture our level of confidence in this estimate (low, medium, high). We rated our estimate as high to medium if we were able to get close to the actual point of origin (ex: intersection of roads) and low if the location description was vague (ex: 6 miles southwest of Sisters Oregon). Our goal was to maximize available geospatial information while allowing users of the data to filter out low-confidence or updated values when a high level of accuracy is needed. The accuracy and completeness of the data improves over time across the three versions, as well as the location description fields available for estimation. In the earliest version (Historical System 1), 29% of coordinates were missing or erroneous but we were able to populate nearly half (49%) of the missing values with estimates taken from the corresponding record in the FPA-FOD database. With limited information, we were only able to manually estimate point of origin for 103 additional values (12%) with the majority of those (89 of the incidents) estimated as low confidence due to limited location information.

In contrast, only 2% of the coordinates were missing or erroneous in the second historical version (Historical System 2) and we were able to populate 45% of the missing values with estimates taken from the corresponding record in the FPA-FOD database. We were able to estimate an additional 26% of missing values with a mix of confidence levels (42 incidents with high confidence, 26 with medium confidence, and 50 with low confidence levels). Finally, only 1.6% of the coordinates were missing or erroneous in the 2014 data with over half of the missing values populated with values taken from the corresponding record in the FPA-FOD database and we were able to estimate all but 2 of the remaining values with a high level of confidence with roughly half requiring a simple swap of latitude and longitude values to correct. Table 9 below summarizes latitude and longitude updates by system and corresponding levels of confidence.

**Table 9:** Latitude/Longitude Updates by System.

Version/File	High	Medium	Low	No Value
Historical 1	447 (438 FOD)	5	89	346
Historical 2	238 (201 FOD)	26	50	130
Current	22 (14 FOD)			2
Total	707	31	139	478

Some of these records are deleted as part of the merging and cleaning process described in section 5 resulting in 98% of incidents with a valid latitude longitude in the final dataset.

### Preparing to merge

The individual fields and values in the incident status reports remained relatively consistent across the three versions, but the underlying data model continued to evolve to adapt to all-hazards management and to capture more detailed information about resources, life safety threat, and management. Our goal when mapping values across the three versions was to maximize continuity while making the historical data forward compatible with the current system. Most of the columns aligned with minimal or no modification. There were several columns that had no equivalent column in the current system. We preserved the ones that had a high fill rate: *Major Problems*, *Observed Fire Behavior*, and *Terrain*. Refer to the column definitions in the ICS-209-PLUS sitrep table^17^.

In addition to the consolidated text fields described in the section above, we added fields to the situation report to align the historical data with the current version or where they added value. For example, the *Acres* field provides a convenient way to compare incident area without having to convert units of measurement. The day of year (DOY) fields (*Discovery DOY and Report DOY)*, *Start Year*, and *Current Year* (CY) support simple querying and analysis without having to manipulate the related timestamps. The script also integrates totals calculated from the related tables into the incident status record. Table 10 summarizes the new values that have been added to the *Incident Status Summary* table.

**Table 10:** New Fields.

Field Name	Description
ACRES	Current size in acres
COMPLEX	True/False indicating incident is part of a fire complex
COMPLEX_NAME	Fire complex name (may or may not be same as *Incident Name*)
CRITICAL_RES_NEEDS_NARR	Critical resources identified for upcoming 12/24/48/72/72 + hours
CURRENT_THREAT_NARR	Current values at risk for upcoming 12/24/48/72/72 + hours
DISCOVERY_DOY	Julian day of the current year for the *Discovery Date* field
EVACUATION_IN_PROGRESS	True/False evacuations in progress
FATALITIES	Current number of reported fatalities
FIRE_EVENT_ID	Unique Identifier for individual fire events
INCIDENT_ID	Unique Identifier for all sitreps grouped under the same incident response
INJURIES	Number of injuries for this reporting period
INJURIES_TO_DATE	Number of injuries to date
NEW_ACRES	Number of acres since the last report
REPORT_DOY	Julian day of the current report
STARTYEAR	Start year of the incident
STR_DAMAGED_RES STR_DESTROYED_RES STR_THREATENED_RES	Total residential structures damaged, destroyed, threatened for the current operational period.
STR_DAMAGED STR_DESTROYED STR_THREATENED	Total structures damaged, destroyed, threatened for the current operational period.
STR_DAMAGED_COMM STR_DESTROYED_COMM STR_THREATENED_COMM	Total commercial structures damaged, destroyed, threatened for the current operational period.
STR_DAMAGED_RES STR_DESTROYED_RES STR_THREATENED_RES	Total residential structures damaged, destroyed, threatened for the current operational period.
TOTAL_AERIAL	Total number of aerial support resources currently assigned to the fire.
TOTAL_PERSONNEL	Total number of personnel resources summed across all agencies

### Purging duplicate and erroneous records

As mentioned above, the historical datasets overlap between 2001 and 2003, and sometimes incident status reports were logged in both systems resulting in duplicate records across the two systems, along with other erroneous records. Many of these records were deleted from the dataset maintained by Karen Short. Rather than deleting each of these records explicitly, we use the records in the Short dataset as a master list (Short1999to2013v2.xlsx).

Any wildfire that does not exist in the master list is removed from the production dataset. Once the cleaning and formatting of the *sitrep* table is complete, the wildfires in the master list are moved to production dataset and the deleted records are archived to a separate deletions file for reference (Table 9). The comparison resulted in the deletion of 527 sitreps from the first historical dataset (3.4%, 57% of these overlapping with Historical System 2) and 3,597 sitreps from the second historical dataset (3.4%).

### Merging and final cleaning

Once each of the individual datasets are refined, historical columns are renamed to align with the corresponding columns in the current version (Table 11) and the individual *Incident Status Summary* tables are concatenated into a single dataset. Unused columns are dropped.

**Table 11:** Columns renamed from Historical Systems 1 & 2.

Current Column Name	Historical System 2 Name	Historical System 1 Name
ADDTNL_COOP_ASSIST_ORG_NARR	COOP_AGENCIES	
CURR_INC_AREA_UOM	AREA	
CURR_INCIDENT_AREA	AREA_MEASUREMENT	
DISCOVERY_DATE	START_DATE	STARTDATE
DISPATCH_PRIORITY	DISPATCH_PRIORITY	DPRIORITY
EST_IM_COST_TO_DATE	COSTS_TO_DATE	ECOSTS
EXPECTED_CONTAINMENT_DATE	EXP_CONTAIN	CDATE
GACC_PRIORITY	GACC_PRIORITY	GPRIORITY
HAZARDS_MATLS_INVOLVMENT_NARR	FUELS	
INC_MGMT_ORG_ABBREV	IMT_TYPE	
INC_MGMT_ORG_DESC	IMT_TYPE_DESC	TEAMTYPE
INCIDENT_COMMANDERS_NARR	IC_NAME	TEAMNAME
INCIDENT_NAME	INCIDENT_NAME	ENAME
INCIDENT_NUMBER	INCIDENT_NUMBER	EVENT_ID
INCTYP_ABBREVIATION	INCTYP_DESC	ITYPE
INCTYP_DESC	INCTYP_DESC	INCTYP_DESC
PCT_CONTAINED_COMPLETED	P_CONTAIN	F_CONTAIN
POO_LATITUDE	LATITUDE	LATDEG + LATMIN
POO_LONGITUDE	LONGITUDE	LONGDEG + LONGMIN
POO_SHORT_LOCATION_DESC	LOCATION	LOCATE
POO_STATE	UN_USTATE	UN_USTATE
PROJ_INC_AREA_UOM	EST_FINAL_AREA	
PROJ_INCIDENT_AREA	AREA_MEASUREMENT	
PROJ_SIG_RES_DEMOB_START_DATE	DEMOBE_START	
PROJECTED_FINAL_IM_COST	EST_FINAL_COSTS	
REMARKS	REMARKS	NARRATIVE
REPORT_TO_DATE	REPORT_DATE + HOUR	REPDATE
SIGNIF_EVENTS_SUMMARY	SIG_EVENT	
**TOTAL_PERSONNEL**	**TOTAL_PERSONNEL**	PERSONNEL
UNIT_OR_OTHER_NARR	UN_UNITID	UN_UNITID

The script then makes a final cleaning and smoothing pass across the records, filling missing values where appropriate and smoothing columns to make them more consistent. The specifics are described below and the columns in the final dataset are described in^17^.

### Filling missing values

Several fields in the dataset are either cumulative or the value, once known, is unlikely to change. We forward filled these fields with the previous known value to minimize gaps and to make sure that these values were propagated to the final report. This was important not just for consistency, but also because these records are used to produce the *Wildfire Incident Summary* table described below. Forward filled fields include: *Acres*, *Estimated Incident Management Costs to Date*, *Fatalities*, *Injuries to Date*, *Latitude*, *Longitude*, *Projected Final Incident Management Costs*, *Total Structures Damaged*, *Total Commercial Structures Damaged*, *Total Residential Structures Damaged*, *Total Structures Destroyed*, *Total Commercial Structures Destroyed*, and *Total Residential Structures Destroyed*.

### Smoothing acres and calculating new acres

Once the forward-filling of acres is complete, we perform a backwards smoothing pass. If the number of *Acres* is downgraded on a subsequent report, we reduce the number of acres on previous reports given that a fire never truly gets smaller, this is likely over-estimation at the time the report was filed. Reducing this was important because we use this value to calculate the *New Acres* field, which is then used to calculate the daily fire spread rate (*Wildfire FSR*) (see Wildfire Incident Summary section below).

### Smoothing cost estimates

The consistency of the cost fields is critical for analysis, even if these fields are subject to bias and real-time information is limited, all records are subject to this bias and it provides a metric for comparing cost across incidents in the research dataset. Both the *Estimated Incident Management Costs to Date* and the *Projected Final Incident Management Cost* fields were sparsely populated with the *Estimated Incident Management Costs to Date* populated only 35% of the time and the *Projected Final Incident Management Cost* only 2% of the time. This field was also particularly prone to data entry error and variations in notation, particularly for estimates in the millions or billions of dollars. When the records were sorted by incident and report date, it was easy to identify instances where someone either left off a digit or added too many for that particular day. Also, as cost increased, sometimes notation changed to simplify data entry (e.g., 1,200,000 becomes 1.2 for $1.2 million dollars). After forward filling the values, we started the cleaning process by manually inspecting all instances where the final reported values were an order of magnitude smaller than the maximum value entered across the reports. We designated the final value based on comparison of trends across the existing reports. We were conservative, only correcting obvious errors. These updates are individually updated in the *cost adjustments* function of the merge script. Once corrections were made to the final reported values, we performed two smoothing passes. We first worked backward from the final cost, adjusting any estimates that were more than 10x larger than the current value by reducing it until it was within the 10x limit. We then worked forward, adjusting any values that were at least 9x smaller the previous estimate until they fell within the 9x limit. When both these passes were complete, if there was no value for the *Projected Final Incident Management Cost*, we defaulted it to the *Estimated Incident Management Costs to Date* on the final report.

### Creating the wildfire incident summary record

The cleaned and merged *Incident Status Summary* table is used to create the *Wildfire Incident Summary* table. This table extracts key elements from the individual sitreps to describe the fire and support high-level analysis across wildfire events. This information includes the cause, discovery date information, final acres, final estimated costs, injuries, fatalities, if evacuations recorded at any point during the fire, and the point of origin (latitude/longitude) for the fire. The summary also includes relevant statistics for structural threat, structures damaged/destroyed and estimates of total personnel and aerial support summed across the fire. We identify peak volumes and corresponding days across the fire including peak personnel, peak aerial, and peak fire spread. Finally, we calculate what we call the *Cessation Date* when the fire grew to within 95% of its final size. This metric is valuable, because the containment date may actually be quite conservative, with incident management teams hesitant to declare a fire contained until there is very limited risk of growth.

### The historical complex associations tables

We use the Short reference data described above to construct a *Complex Associations Table* for the historical datasets. This information is limited. It clusters incident names and numbers under associated fire complex and tallies the number of sitreps related to each. This table is described in Table 12 below.

**Table 12:** Historical Complex Associations Table.

Column Name	Description
COMPLEX_INCIDENT_ID	Incident Id value for fire complex
COMPLEX_NAME	Final name for fire complex
INC_MGMT_NUM_SITREPS	Number of sitreps related to Incident Number/Incident Name combination
INCIDENT_NUMBER	Incident number for each incident incorporated into complex

## Data Records

The initial release of the ICS-209-PLUS dataset spans sixteen years from 1999 to 2014 and contains 124,411 *Incident Status Summary* reports for 25,083 thousand all-hazard incidents. The dominant hazard in the dataset is wildland fire (98.3%) with the remaining 1.7% spread across other hazards. The number of incidents is lower overall prior to 2005 mandate, but contains roughly the same distribution of fire and non-fire incidents as in subsequent years. Given the dominance of wildfire in the dataset, we created three tables specifically for wildland fire analysis: a Wildfire-specific *Incident Status Summary* table with just the wildfire sitreps, a *Wildfire Incident Summary* table with key values related for each fire, and a *Complex Associations* table (Historical only 1999 to 2013). Table 13 below summarizes each of the tables in the ICS-209-PLUS dataset and the number of records contained in each.

**Table 13:** Tables in Production Dataset.

Dataset Table Name	Description	# Records
ics209-plus_sitreps_1999to2014 (ics209-plus-allhazards.zip)	All-hazards dataset	124,411
ics209-plus-wf_sitreps_1999to2014 (ics209-plus-wildfire.zip)	All wildfire and prescribed burn sitreps	120,825
ics209-plus-wf_incidents_1999to2014 (ics209-plus-wildfire.zip)	Incident level summary for all wildfire incidents and prescribed burns	24,608
ics209-plus-wf_complex_assocs_1999to2013 (ics209-plus-wildfire.zip)	Summary of wildfire complexes for historical dataset	1,905
ics209-plus-wf_complex_assocs_2014 (ics209-plus-wildfire.zip)	Summary of wildfire complexes for current ics209 system	70
ics209_sitreps_deleted_hist1_1999to2002 (ics209-plus-wildfire.zip)	Deleted records from hist1	527
Ics209_sitreps_deleted_hist2_2001to2013 (ics209-plus-wildfire.zip)	Deleted records from hist2	3,597

All files used to produce the dataset are packaged with yearly database table files as part of the ics209-plus-source.zip file. These input files and directory locations are summarized in Table 14 below. Table definition and field fill rates are also available online in the ics209-pluse-reference.zip file (Table 15 below). The dataset, input, source, and reference files can be found online at figshare^17^.

**Table 14:** Input Tables Used to Produce Dataset.

Input Table Name	Description & Location
FOD_CPLX_REF_2014.xlsx	Reference for all Fire Complexes in the FPA/FOD Database and ICS-209-PLUS Dataset and the individual Fire Events related to those Fire Complexes (data/raw/excel/fod)
FOD_JOIN_{timestamp}.xlsx	Latest version of Dataset linking FPA/FOD Dataset with ICS-209-PLUS. *Linked based on ICS_209_PLUS_INCIDENT_ID (data/raw/excel/fod)
Latitude/Longitude Update Files • legacy_cleaned_ll-fod.csv • historical_cleaned_ll-fod.csv • 2014_cleaned_ll-fod.csv	Estimate or replacement values for all latitude and longitude values in the ICS-209 dataset that did not fall within bound of US or US territories, along with confidence estimates. (data/raw/latlong_clean)
Short1999to2013v2.xlsx	Short Master List for Historical Wildfires. Only Historical Wildfires in this list are in production dataset. (data/raw/excel)

**Table 15:** Reference Tables.

Input Table Name	Description
ics209-plus-wf-incident_definitions.xlsx	Summary of column and fill rates for the Wildfire Incident Summary Table
ics209-plus_sitrep_definitions.xlsx	Summary of column and fill rates for the Daily Situation Report Table. Separate columns for all-hazards versus wildfire datasets.
sit209_lookup_codes_definitions.xlsx	Summary of standard values for all categorical fields in the ICS-209 dataset

## Technical Validation

It is important to note that the ICS-209-PLUS represents a small but important subset of wildfires (1–2%). The trends presented in this technical validation are for large incidents only and not meant to be interpreted as holding for wildfires in general.

### Wildfire distribution

**Fig. 1:**
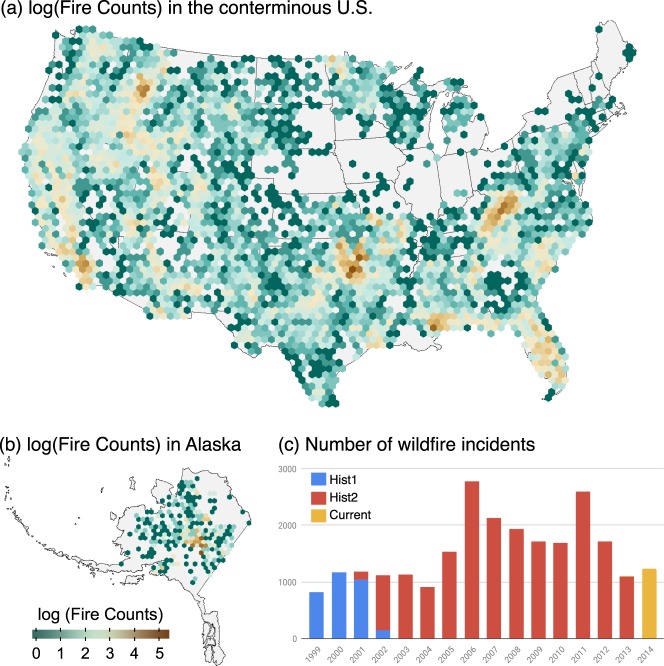
The log number of incidents within a 50-km hexagonal grid cell for the U.S.

Wildfires requiring the use of ICS are spread across the interior of Alaska and continental U.S. with higher concentrations in parts of California, the Northern Rockies, Northern Forests, and parts of the Southeastern U.S. There were a small number of fires in Hawaii and Puerto Rico (not shown). Fire is notably limited in areas of the Central Midwest and Northeastern US. The spatial distribution of incidents for the continental U.S. and Alaska with the total number of incidents by year is shown in Fig. 1 below.

### Trends across key variables

**Fig. 2:**
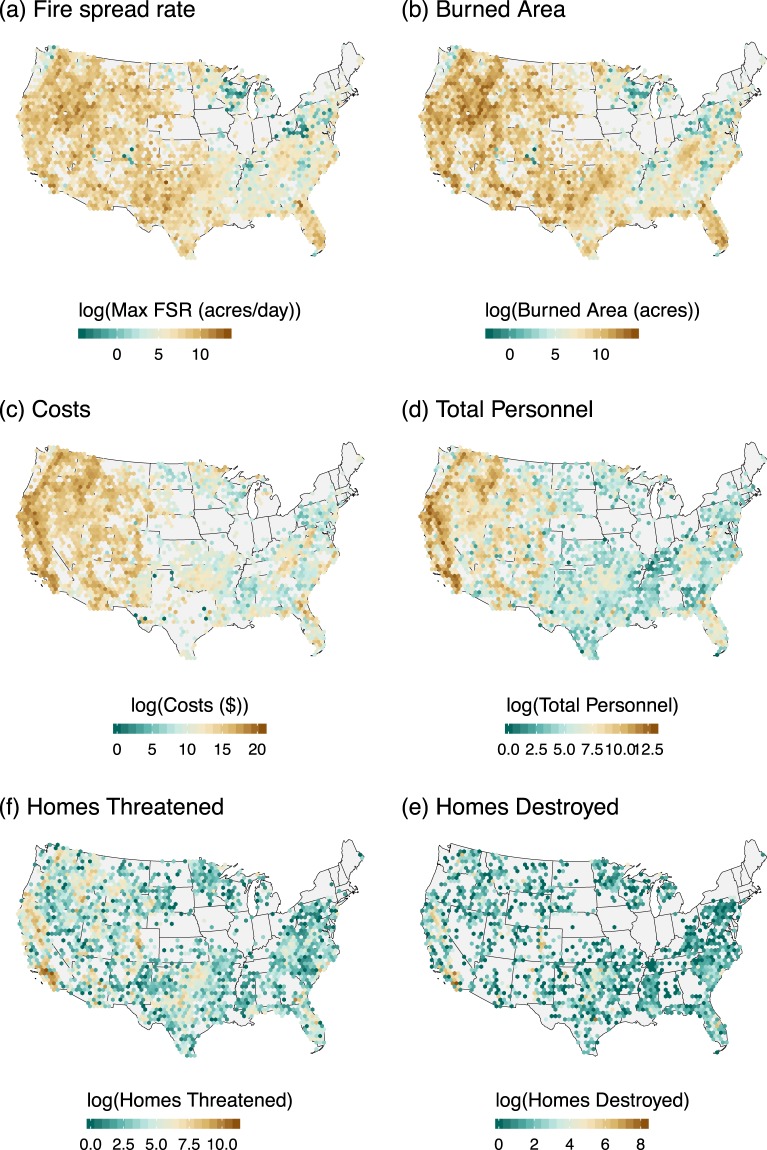
Spatial distribution of key variables for Continental United States (CONUS).

We examined the national patterns in six variables within the dataset: maximum fire spread rate, burned area, projected final costs, total personnel (total personnel summed across sitreps), maximum number of homes threatened, and the number of homes destroyed (Fig. 2). Not surprisingly, the fastest fires were located in the northern Great Basin area, along the border of Nevada and Idaho, landscapes that are dominated by cheatgrass fuels. Additionally, the west experienced larger, faster fires requiring more resources and resulting in higher suppression costs. Suppression costs are an order of magnitude lower on average in the east versus the west, but that societally impactful fires are not limited to the West (Fig. 2ef). Smaller fires in the east threaten and destroy large numbers of homes. The allocation of firefighting personnel is heaviest in California and the Pacific Northwest, with California committing the heaviest resources regionally, which may help mitigate the number of homes damaged or destroyed given the population density and large number of homes threatened across these fire-prone landscapes, except in the hottest and driest areas portion of Southern California. Large wildfires have a presence in all but key areas of the midwest and northeast. In the Appalachian mountain range, the influence of human ignitions has led, in recent years, to large of areas burned, requiring management of an incident command team that is apparent in this dataset. More research is needed to understand the factors at play in these fires outside of the scope of this work.

Summary statistics were generated across the national Geographic Area Coordination Centers (GACCs) for the six key metrics (Table 16). Most notably in the conterminous U.S., the Great Basin experienced the fastest fires, with an average maximum fire spread rate of 3,147 acres/day, yet, Alaska reported the fastest average maximum fire spread rate across all GACCs (5,620 acres/day) and largest average wildfire (22,738 acres). The average fire size in the southern GACC (southeastern U.S.) was 850 acres, or roughly 85% smaller than average fire size compared to all other GACCs. Though wildfires were substantially smaller in the southern GACC, it experienced the second highest structures threatened (136,592) and destroyed (9,555). The state of California consists of the South Ops and North Ops GACCs, and, when combined, represent 41% of the total suppression costs, 48% of total personnel employed, 45% of all structures threatened, 40% of all structures destroyed, yet only 11% of the total burned area across all GACCs.

**Table 16:** Summary Statistics Table.

GACCs	Fire Count	Total Burned Area (acres)	Mean Fire Spread Rate	Mean Max Fire Spread Rate	Total Suppression Costs ($)	Total Personnel	Total Structures Threatened	Total Structures Destroyed
Alaska	549	12,483,035	22,738	5,620	$ 472,189,687	416,647	20,755	381
Eastern	2,262	1,190,622	526	302	$ 99,213,391	136,859	14,905	1,578
Great Basin	2,919	18,484,346	6,332	3,147	$ 1,898,282,148	2,087,367	97,060	1,707
North Ops	870	3,592,381	4,129	1,356	$ 3,908,975,978	5,131,034	101,165	3,724
Northern Rockies	1,556	6,164,345	3,962	1,607	$ 1,560,623,656	1,770,149	37,199	1,341
Northwest	1,234	9,102,733	7,377	3,093	$ 2,829,753,743	3,152,001	67,880	1,906
Rocky Mountain	1,299	4,446,751	3,423	1,878	$ 757,662,495	849,302	96,230	3,749
South Ops	1,712	4,897,332	2,861	1,198	$ 2,754,595,274	4,931,373	400,743	12,483
Southern	9,394	7,987,737	850	525	$ 488,948,676	802,946	136,592	9,555
Southwest	2,177	12,984,638	5,964	2,888	$ 1,386,524,283	1,283,577	101,204	3,304
Totals	23,972	81,333,921	5,816	2,161	16,156,769,332	20,561,254	1,073,732	39,728

(refer to Supplementary Tables 1 and 2 for summary statistics across level 1 Ecoregions^25^ and state administrative boundaries)

### National interagency fire center (NIFC) comparison

We compared the ICS-209-PLUS dataset with annual fire statistics provided by the National Interagency Fire Center (www.nifc.gov) across the same time period. The ICS-209-PLUS dataset captured approximately 2% (range 0.6% to 3.5% annually) of the population of wildfires, accounting for approximately 80% of the acres burned (range 53% to 98% of acres burned annually) and 79% of the suppression costs (range 51% to 140% of costs annually). These numbers are roughly in line with what you would expect, but the annual variability indicates that there are still significant outliers in the values that are skewing the results.

### Case study

#### The 2013 california rim fire

The California Rim fire started on August 17th, 2013 in a remote canyon of the Stanislaus National Forest as the result of an illegal campfire. The fire was discovered when it was approximately 40 acres in size but due to drought, extreme weather conditions, inaccessible terrain, and erratic winds, the fire grew to over 10,000 acres in just 36 hours and grew to over 100,000 acres in the first four days. It was not declared fully contained until October 24th, 2013, reaching a final size of 257,314 acres, making it the third largest fire in California history at the time. At its peak, more than 5000 firefighting resources were assigned to the fire, and it cost $127.35 million dollars to suppress (https://inciweb.nwcg.gov/incident/5595/). It resulted in 10 injuries and no fatalities. At its peak, the fire threatened over 5,000 structures and resulted in the evacuation of over 15,000 people, destroying over a hundred structures including eleven residences.

**Fig. 3:**
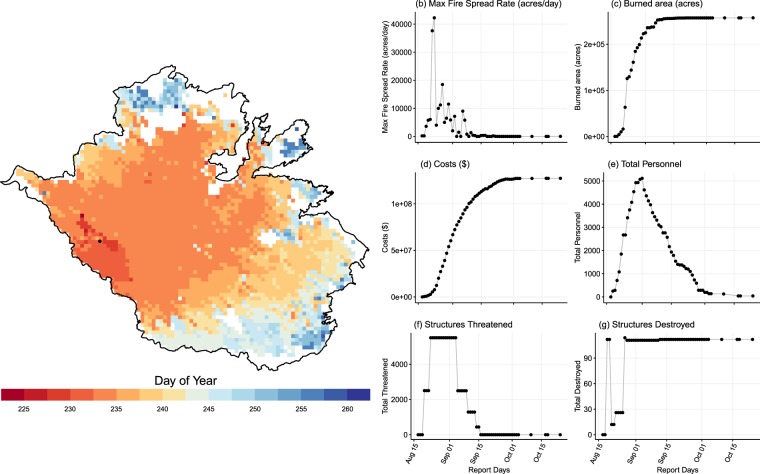
Key metrics illustrating the daily reporting for the Rim Fire via the ICS-209 daily reports.

There are 100 situation reports for the Rim Fire spanning fire discovery on August 17th at 3:25 pm through the declared containment on October 24th. Looking across key metrics on the 209 reports, one can see the fire narrative play out across the situation reports (Fig. 3). There is a close relationship across the peaks of these variables. There is a clear lag in the reporting of burned area, which may reflect delays in finalizing estimates, particularly in the most volatile phase of large and fast fires. The fire is not estimated at 10,000 acres until the third day and does not reach 100,000 acres until the morning of the seventh day. The acreage stabilizes as the growth slows on September 6th. The estimated final incident management costs rise steeply during the active growth phase of the fire and level out at $46 million dollars, less than half the final estimated incident management cost. This example illustrates that estimates are likely to be conservative, particularly on large, fast-moving events with estimates based on what is known in the moment rather than a clear accounting of final cost. It may take months for the final numbers to be tallied on large-scale responses. The allocation of firefighting personnel ramps quickly to just over 5,000 on September 1st, fourteen days into the fire and then begins to taper down equally quickly. The number of homes threatened rises quickly, with the first threat reported as 25 structures at 6 pm on August 19th. This value jumps to 2,505 at 7am the following morning with a total of seven structures destroyed including two residences, and again on August 23rd to 4,503 structures at 6am and 5,503 structures at 7:30 pm, coinciding with issuing of new evacuation advisories.

The values logged for both total personnel and structures threatened may represent the most knowable information in-the-moment. The tracking of personnel happens in real-time during a shift and concrete estimates of homes and areas threatened by a wildfire are integral to incident management decisions. It is also likely that there is a lag between resource needs and the deployment of new firefighting personnel. We hypothesize that the first report of structures threatened may serve as an accurate indicator of the onset of social disruption and that the growth and levelling out of personnel indicates that the fire is in its most acute and socially disruptive phase with the onset of demobilization of resources indicating the easing of the threat posed by a fire. In this case, the first structural threat is recorded at 6 pm on August 19th and jumps to over 2,500 the following evening. This figure reaches it peak at over 5,000 structures approximately 48 hours later on August 22nd, where it remains for thirteen days. The number of personnel steadily increases until September 1st when resources begin to drop.

The perimeter map (Fig. 3a) visually depicts the rapid growth of the fire. The black dot at the bottom left is the Point of Origin Latitude/Longitude from the ICS 209 report. The perimeter outline is taken from the MTBS dataset^24^ and the fire progression is constructed using the MODIS burned area data^26^. Because the MODIS data records the last burn detection for each pixel, the maximum growth is recorded on day five of the fire, which is earlier than the formal reporting of this same acreage on the sitrep. This highlights two important points. Knowing that there is a likely delay in the reporting of acres burned on the sitreps, we may be able to make adjustments to these values as part of any analysis. Additionally, knowing the strengths and weaknesses of the values on the 209 and the inherent biases, it could potentially be combined with other data sources such as remote sensing to correct these biases that connects the physical measures of a wildfire with important measures of social disruption and firefighting resources.

## Usage Notes

As wildfire activity has increased in the U.S. over the past several decades^5–8^, the ICS-209-PLUS dataset offers a unique opportunity to explore the costs and consequences of the nation’s major wildfire events. This dataset captures a unique perspective that complements other important sources of information on wildfires, from government compiled databases (FPA-FOD^23^) to satellite-derived detections of active fire or burned area (i.e., Landsat, MODIS, VIIRS^24,26,27^).

This dataset captures critical details about an important subset of wildfires in the United States, those that require the establishment of an incident command team. Although these large, serious fires account of only 2% of wildfires, they account for approximately 80% of suppression costs. The daily situation reports capture the best in-the-moment information across the changing characteristics of the fire, environmental conditions such as weather and terrain, incident response, and the built and natural values at risk. As the magnitude of wildfires grow and we continue to expand our reach into the wildland urban interface (WUI), the values captured across this important population of fires provides a unique opportunity to understand the relationship between changing fire regimes, incident response, and the social decisions we are making in relation to these risks.

The first revision of this dataset accomplishes several key objectives. It aligns the underlying data model across the three versions of the system from 1999 to 2014, making it possible to effectively compare values across these three versions of the ICS-209 reporting system for longer timeframe than previously possible with minimal effort. Substantial cleaning efforts and the filling of missing values allow for more accurate assessment across the reports and to analyze trends more effectively. Additionally, the code used to create this dataset is open source and can easily be extended to process and add subsequent years to the existing dataset. The effort here represents something that could be taken on at a large scale, to make the data more publicly available on a natural hazard that increasingly threatens lives and infrastructure. More work is needed to streamline the process so that new data can be processed and downloaded without manual effort. Additionally, more work is needed to streamline the publication process. The data from 2015 forward needs to be updated so that it is a complete and accurate record of daily situation records with associated record for personnel resources, structural threat, and life safety. Finally, the data is published after it is finalized at the end of the year. There is potential to build tools that capture these ‘in-the-moment’ reports in real-time, allowing new questions to be asked in real-time and for this data to be integrated with other sources such as satellite-derived data.

One of the longstanding barriers to using the ICS-209 has been the data quality and lack of alignment across the three reporting systems. Like most human-generated, observational datasets, the fields are free-form and difficult to wrangle. Our goal was to strike a balance between manual inspection/cleaning where necessary and programmatic data cleaning. As highlighted above, we focused manual efforts on high-value fields like latitude/longitude and cost where we felt cleaning would provide the highest initial return. We also automated the standardization of values across the reporting systems and auto-filled empty values wherever it made sense. Moving forward, the data will continue to be messy and data duplication and field-level errors are impossible to plan for and eradicate. As highlighted above, better mechanisms for crowd-sourcing the reporting of errors and automating updates could greatly improve the integrity of the data, particularly as this dataset is adopted for widespread use.

Our hope is that making this data available will lead to cross-sector and cross-discipline work that leads to greater understanding of our nation’s wildfire trends and the consequences of those changing trends. Understanding the causes and consequences of wildfire is a complex task requiring expertise across disciplines and potentially benefiting those in the forefront of fire management, climate science, natural hazards research, policy making, planning and development. The ICS-209-PLUS data provides an important level of detail that can be used in parallel with other sources of information, filling in gaps and providing a more complete and nuanced picture of the relationship between characteristics of wildfire, incident response, and the causes and consequences of threatening wildfires in the national landscape. There is potential to address a critical informational need as we work to understand trends and address the impacts and consequences of fire in an evolving physical and social landscape. This dataset has a great future research benefit, particularly if current limitations are addressed effectively through community-wide efforts to keep improving on this rich dataset in an open science framework.
